# Research highlight: Impact of COVID-19 Disease Control Committee (CDCC) policies on prevention of the disease using Bayes network inference in west of Iran

**DOI:** 10.1186/s12889-024-17901-7

**Published:** 2024-03-18

The COVID-19 pandemic has had a global impact, with millions of infections and deaths. Managing the virus's spread has been difficult, with different countries implementing diverse strategies. These strategies include quarantine, social distancing, mask-wearing, hand hygiene, travel restrictions, and vaccination.

This research focuses on the COVID-19 Disease Control Committee (CDCC) in Hamadan province, Iran, and their efforts to control and lessen the virus's risk. The study aimed to assess the impact of the CDCC's policies and plans on controlling and lessening the coronavirus risk.

This observational study was carried out from April to August 2021, before the fifth wave of the COVID-19 pandemic hit Hamadan. The data for the study were collected from three sources: CDCC session reports, information from periodic surveys conducted by the Primary Health Care directory in Hamadan province, and expert panel opinion. The study included a review of the committee's reports and decision-making process over five months.

The study's results revealed that several strategies effectively reduced the virus's spread, such as vaccination, limiting gatherings, social distancing, mask-wearing, job closure, travel restriction, and personal hygiene. The study discovered that as the implementation of these strategies increased, the risk of catching the disease significantly decreased. For example, vaccination was the most crucial factor in controlling the virus's spread. If the intensity of these policies increased by 10%, the COVID-19 risk decreased from 42.06% to 38.85%.

The study concludes that while selecting the best policy to reduce and control diseases is challenging, in the case of contagious pandemics like COVID-19, emphasis on vaccination, avoiding gatherings, physical distancing, and mask-wearing can significantly lessen the risk. The study also highlights the importance of a comprehensive information registration system for continually evaluating different policies and scenarios to control unknown diseases, especially in areas where strict quarantine is not feasible due to economic and technological constraints.

This study offers valuable insights into the effectiveness of various strategies in controlling COVID-19 spread and can inform future decisions in managing such pandemics.

*The following summaries of hand-selected papers were generated by Springer Nature's artificial intelligence tool and revised by a subject matter expert to meet Springer Nature's standards.*

